# Supplementary material for: Comparison of lean mass indices as predictors of mortality in incident peritoneal dialysis patients
Source: PLoS One. 2021 Jul 22;16(7):e0254942. doi: 10.1371/journal.pone.0254942 (PMC8297877; doi:10.1371/journal.pone.0254942)
Supplement: S1 Table — (DOCX) [file pone.0254942.s001.docx]

**S1 Table. Comparison of clinical characteristics between included and excluded participants at the time of peritoneal dialysis initiation**

|  | **Total (n = 694)** | **Excluded participants**  **(n = 166)** | **Included participants**  **(n = 528)** | ***P*-value*** |
| --- | --- | --- | --- | --- |
| Age (years) | 54.1 ± 14.1 | 55.9 ± 15.8 | 53.5 ± 13.5 | 0.079 |
| Sex (male) | 382 (55.0%) | 96 (57.8%) | 286 (54.2%) | 0.408 |
| Body mass index (kg/m^2^) | 23.5 ± 3.3 | 23.3 ± 3.3 | 23.6 ± 3.3 | 0.263 |
| RRF (mL⋅min^-1^⋅1.73 m^-2^) | 3.9 ± 3.4 | 3.6 ± 3.1 | 4.0 ± 3.5 | 0.160 |
| Serum albumin (g/dL) | 3.43 ± 0.56 | 3.41 ± 0.43 | 3.48 ± 0.55 | 0.173 |
| C-reactive protein (mg/dL) | 0.80 ± 1.99 | 0.79 ± 1.43 | 0.70 ± 1.70 | 0.553 |
| Modality (APD) | 105 (15.1%) | 18 (10.8%) | 87 (16.5%) | 0.083 |
| Edema index | 0.368 ± 0.030 | 0.370 ± 0.026 | 0.368 ± 0.030 | 0.495 |
| Weekly Kt/Vurea | 2.37 ± 0.74 | 2.28 ± 0.71 | 2.39 ± 0.74 | 0.121 |
| Follow-up duration (mon) | 48.4 ± 41.5 | 51.2 ± 48.1 | 53.3 ± 41.0 | 0.573 |
| Davies risk index |  |  |  | 0.599 |
| Low | 233 (33.6%) | 54 (32.5%) | 179 (33.9%) |  |
| Intermediate | 418 (60.2%) | 99 (59.6%) | 319 (60.4%) |  |
| High | 43 (6.2%) | 13 (7.8%) | 30 (5.7%) |  |

Data are expressed as numbers (percentages) for categorical variables and as median ± standard deviation for continuous variables.

**P-*values were tested using the Student’s *t*-test for continuous variables and Pearson’s χ^2^ or Fisher’s exact test for categorical variables.

Abbreviations: RRF, residual renal function; APD, automated peritoneal dialysis.
