# Supplementary material for: Comparison of lean mass indices as predictors of mortality in incident peritoneal dialysis patients
Source: PLoS One. 2021 Jul 22;16(7):e0254942. doi: 10.1371/journal.pone.0254942 (PMC8297877; doi:10.1371/journal.pone.0254942)
Supplement: S2 Table — (DOCX) [file pone.0254942.s002.docx]

**S2 Table. Univariate and multivariate hazard ratios using competing risk model according to various indices**

| **Independent variables** | **Univariate** | |  | **Multivariate** | |  |
| --- | --- | --- | --- | --- | --- | --- |
|  | **Hazard ratio (95% CI)** | ***P*-value** |  | **Hazard ratio (95% CI)** | ***P*-value*** | |
| Men (per increase 1 unit) |  |  |  |  |  | |
| ALM | 0.913 (0.862–0.966) | 0.002 |  | 0.988 (0.926–1.054) | 0.713 | |
| ALM/Ht^2^ | 0.829 (0.691–0.995) | 0.044 |  | 0.951 (0.785–1.151) | 0.606 | |
| ALM/BW | 0.976 (0.920–1.034) | 0.410 |  | 1.004 (0.950–1.062) | 0.881 | |
| ALM/BMI | 0.110 (0.021–0.577) | 0.009 |  | 1.228 (0.201–7.485) | 0.824 | |
| LTLM ratio | 0.021 (0.003–0.151) | <0.001 |  | 0.091 (0.008–1.050) | 0.055 | |
| Women (per increase 1 unit) |  |  |  |  |  | |
| ALM | 0.920 (0.846–1.000) | 0.051 |  | 0.957 (0.871–1.052) | 0.362 | |
| ALM/Ht^2^ | 0.852 (0.690–1.052) | 0.137 |  | 0.822 (0.635–1.062) | 0.134 | |
| ALM/BW | 0.930 (0.880–0.982) | 0.009 |  | 0.936 (0.870–1.007) | 0.077 | |
| ALM/BMI | 0.040 (0.005–0.343) | 0.003 |  | 0.177 (0.011–2.780) | 0.218 | |
| LTLM ratio | 0.014 (0.001–0.145) | <0.001 |  | 0.055 (0.004–0.858) | 0.039 | |

*Multivariable analysis was adjusted for age, the Davies risk index, weekly Kt/Vurea, residual renal function, C-reactive protein, and edema index.

Abbreviations: CI, confidence interval; ALM, appendicular lean mass; ALM/Ht^2^, appendicular lean mass per height squared; ALM/BW, appendicular lean mass per body weight; ALM/BMI, appendicular lean mass per body mass index; LTLM, limb/trunk lean mass ratio.
